# Nanoparticles in Plant Cryopreservation: Effects on Genetic Stability, Metabolic Profiles, and Structural Integrity in Bleeding Heart (Papaveraceae) Cultivars

**DOI:** 10.2147/NSA.S485428

**Published:** 2025-02-17

**Authors:** Dariusz Kulus, Alicja Tymoszuk, Alicja Kulpińska, Bożena Dębska, Agata Michalska, Julita Nowakowska, Dorota Wichrowska, Jacek Wojnarowicz, Urszula Szałaj

**Affiliations:** 1 Laboratory of Horticulture, Department of Biotechnology, Faculty of Agriculture and Biotechnology, Bydgoszcz University of Science and Technology, Bydgoszcz, Poland; 2 Department of Biogeochemistry and Soil Science, Faculty of Agriculture and Biotechnology, Bydgoszcz University of Science and Technology, Bydgoszcz, Poland; 3 Imaging Laboratory, Faculty of Biology, University of Warsaw, Warsaw, Poland; 4 Department of Microbiology and Food Technology, Faculty of Agriculture and Biotechnology, Bydgoszcz University of Science and Technology, Bydgoszcz, Poland; 5 Laboratory of Nanostructures, Institute of High Pressure Physics, Polish Academy of Sciences, Warsaw, Poland

**Keywords:** antioxidant capacity, genotyping, HPLC, *Lamprocapnos spectabilis* (L.) Fukuhara, phenolic acids, phenylpropanoid pathway, TEM

## Abstract

**Purpose:**

Studying the role of nanoparticles in plant cryopreservation is essential for developing innovative methods to conserve plant genetic resources amid environmental challenges. This research investigated the effects of gold (AuNPs), silver (AgNPs), and zinc oxide (ZnONPs) nanoparticles on the structural integrity, genetic stability, and metabolic activity of cryopreserved plant materials with medicinal properties.

**Methods:**

Shoot tips from two bleeding heart (*Lamprocapnos spectabilis* (L). Fukuhara) cultivars, ‘Gold Heart’ and ‘Valentine’, were cryopreserved using the encapsulation-vitrification technique, with nanoparticles added at concentrations of 5 or 15 ppm during either the preculture phase or the alginate bead matrix formation. Post-recovery, the plants underwent histological, molecular, and biochemical analyses.

**Results:**

Electron microscopy observations of LN-derived plant material confirmed the production of micro-morpho-structurally stable cells. It was found that nanoparticles could penetrate the cell and accumulate in its various compartments, including the nucleus. As for the genetic analysis, SCoT markers identified polymorphisms in 11.5% of ‘Gold Heart’ plants, while RAPDs detected mutations in 1.9% of ‘Valentine’ specimens. Analysis of Molecular Variance (AMOVA) indicated that in the ‘Valentine’ cultivar, all genetic variation detected was within populations and not significantly affected by nanoparticle treatments. In ‘Gold Heart’, the majority (94%) of genetic variation detected was within populations, while 6% was attributed to nanoparticle treatments (mostly the application of 15 ppm ZnONPs). The application of nanoparticles significantly influenced the metabolic profile of bleeding heart plants, particularly affecting the synthesis of phenolic acids and aldehydes, as well as the antioxidant mechanisms in both ‘Gold Heart’ and ‘Valentine’ cultivars. The content of proteins was altered in ‘Gold Heart’ plants but not in ‘Valentine’.

**Conclusion:**

The results suggest that different types and concentrations of NPs have varying effects on the production of specific metabolites, which could be harnessed to modulate plant secondary metabolism for desired pharmacological outcomes.

## Introduction

*Lamprocapnos spectabilis* (L). Fukuhara (formerly *Dicentra spectabilis* Lem)., commonly known as Bleeding Heart, is a herbaceous perennial plant belonging to the Papaveraceae family.^1^ This species is characterized by its unique inflorescence structure, featuring pendulous racemes of distinctive, bilaterally symmetrical, heart-shaped flowers that typically range in colour from red to white. *Lamprocapnos spectabilis* is widely cultivated for its ornamental value in horticulture. Its attractive, fern-like foliage emerges early in the growing season, making it a popular choice among florists and gardeners.^2^ Even though bleeding heart is primarily recognized for its ornamental values, it also has applications in medicine. Some historical medicinal uses of bleeding heart include the treatment of skin irritations and as a mild diuretic.^3^ The presence of alkaloids and isoquinoline derivatives, such as protoberberines, has been reported in this species. These compounds may have potential pharmacological properties, for example in cancer treatment, as antidepressants, and in skin care.^4–6^ Consequently, bleeding heart is an interesting plant material in biotechnological research. One of the current mainstream directions in science includes the effect of nanomaterials on plants.

The integration of nanoparticles (NPs) into plant biotechnology has opened a new era of innovation in agriculture, horticulture, and crop production. Nanoparticles, characterized by their tiny size (below 100 nm) and unique physicochemical properties, have been used to revolutionize various aspects of plant biology.^7^ The incorporation of nanoparticles in biotechnology holds the promise of increasing crop yields, reducing resource wastage, and promoting sustainable farming practices, but it also raises questions about their safety and potential adverse impacts on plants.^8^

The impact of nanoparticles on plants has been broadly studied.^9^ NPs can penetrate plant tissues and cells, leading to alterations in cellular morphology and structure. For example, polyvinyl chloride nanoparticles can interact with lipid bilayers in *Arabidopsis thaliana* L. root cells, leading to reduced density and changes in fluidity and membrane thickness.^10^ This can result in increased permeability of cell membranes, affecting nutrient uptake, ion transport, and overall cell function. Moreover, NPs can accumulate in organelles such as chloroplasts and mitochondria, affecting their structure and function.^11^ For example, in chloroplasts, NPs may disrupt thylakoid membranes, interfere with photosynthetic processes, and lead to reduced chlorophyll content and impaired photosynthesis as observed in *Vicia faba* L. after silver nanoparticles treatment.^12^ The final effect depends on NPs concentration, size, and type but also plant species and even cultivar.^11^

As for genotoxic effects, NPs can induce damage at the genetic level in plants through several mechanisms. Since nanoparticles can penetrate plant cells, they can directly interact with genetic material.^13^ NPs can cause DNA damage through oxidative stress. When NPs enter plant cells, they can generate harmful reactive oxygen species (ROS) through redox reactions. ROS can then oxidize DNA bases, induce DNA strand breaks, and cause DNA cross-linking, all contributing to genotoxicity.^14^ Furthermore, NPs may interfere with DNA repair mechanisms in plants or affect gene expression patterns by interacting with transcription factors or epigenetic modifiers.^15^ Additionally, some NPs have been found to induce chromosomal aberrations. This includes changes in chromosome structure, such as chromosome breaks, stickiness of chromosomes, nuclear notch, and clumped chromosomes,^16^ which can disrupt the normal functioning of genes and lead to adverse effects on plant growth and development. For these reasons, silver nanoparticles (AgNPs) have been successfully used in the mutation breeding of ornamental plants, such as *Chrysanthemum × morifolium* (Ramat). Hemsl.^17^

Finally, nanoparticles can affect the metabolite profile of plants. They can either enhance or inhibit metabolic pathways, depending on numerous factors such as nanoparticle size, concentration, and treatment duration. Some studies have shown that certain types of NPs, such as silver, zinc oxide (ZnONPs), and titanium dioxide nanoparticles (TiO_2_NPs), can stimulate the biosynthesis of secondary metabolites (phenolics, flavonoids, terpenoids, and phytoalexins), which play crucial roles in plant defense mechanisms against biotic and abiotic stresses but are also valuable in the pharmaceutical industry.^18–20^ Likewise, silver nanoparticles enhanced the content of proteins and carbohydrates in *Pisum sativum* L. seeds.^21^ As for in vitro germinated seedlings of *Solanum lycopersicum* L. and *Brassica oleracea* L., a decrease in the content of chlorophylls, carotenoids (in both species), and anthocyanins (in kale) due to silver nanoparticle-treatment was reported.^22^

The integration of nanotechnology into cryobiology research opens new possibilities in germplasm protection. Cryopreservation in liquid nitrogen (LN, −196°C) is considered the safest method for long-term storage of cells and tissues. This approach enables the protection of genetic diversity, conservation of endangered species, and supports agriculture by preserving plant cultivars for future use.^23^ Nonetheless, challenges remain in cryopreservation including the risk of cellular damage due to ice formation, the potential toxicity of cryoprotectants, and the need for optimized protocols that ensure the long-term viability of preserved cells. Some of these issues can be addressed by NPs. Nanoparticles were successfully applied in low-temperature storage of animal cells and gametes. They can enhance the thermal conductivity of cryoprotectant agents, significantly improve nucleation, and lower devitrification temperatures in cryoprotectant solutions. Additionally, suitable NPs may protect DNA in cells and strengthen the adhesion between cells and scaffolds.^24^ Moreover, polymeric nanoparticles effectively deliver cryoprotectants and other protective agents, especially those that cannot penetrate cell membranes, into mammalian cells.^25^ It was also reported that the survival rate of LN-derived plant material can be enhanced through the integration of nanoparticles into the cryopreservation protocol. The addition of gold nanoparticles (AuNPs) into the protective alginate matrix increased the viability of bleeding heart shoot tips by 20%.^26^ To date, however, there have been no studies comparing the effect of various nanoparticle types on the genetic and structural integrity of cryopreservation-derived plant material, as well as its detailed metabolic profile. Usually, storage in LN does not affect the metabolic or genetic stability of plants, although sometimes minor changes at the cellular, genetic and metabolome levels can be found.^27^ As the field advances, critical considerations regarding nanoparticle safety, their genotoxic effects, and regulatory effects in plants must be addressed to ensure responsible use in plant biotechnology. Therefore, the aim of this study was to investigate the effect of gold, silver and zinc oxide NPs applied during the cryopreservation procedure on the structural integrity, genetic stability and metabolic activity of LN-derived plant material.

## Materials and Methods

### Plant Material and Cryopreservation Procedure

In vitro-derived shoot tips of bleeding heart (*Lamprocapnos spectabilis* (L). Fukuhara) ‘Gold Heart’ and ‘Valentine’ (1.0–2.0 mm long) were used as explants. The plants were obtained from the gene bank of the Laboratory of Horticulture, Bydgoszcz University of Science and Technology, Poland. The plants were identified and authenticated by dr. Tomasz Stosik, member of the Polish Botanical Society (PBS). Voucher specimens (no. 430 for ‘Gold Heart’ and no. 431 for ‘Valentine’) were deposited in the Herbarium of the Department of Botany, Ecology and Landscape Architecture, Bydgoszcz University of Science and Technology, Poland. The cryopreservation procedure described in detail by Kulus et al^28^ consisted of the following steps: preculture, encapsulation, dehydration/vitrification, low-temperature storage (LN), rewarming, recovery, and acclimatization.

### Experiment I – NPs in the Preculture Medium

Single-node explants were cultured for one week on a Murashige and Skoog (MS)^29^ medium containing 9% sucrose, 4.65 μM kinetin, and 10 µM abscisic acid (all plant growth regulators provided by Sigma-Aldrich, Darmstadt, Germany). Each culture vessel (glass jar) contained ten explants and was filled with 30 mL of medium, which was sterilized at 121^°^C, 0.1 MPa, for 20 min.

Suspensions of silver (6 nm in diameter; *v*/*v*), gold (6 nm; *v*/*v*), or zinc oxide (25 nm; *w*/*v*) nanoparticles, at concentrations of 5 and 15 ppm, were evenly distributed on the culture medium surface through a sterile filter immediately after explant inoculation (2 mL per jar). The control group comprised non-treated explants. The detailed description of the synthesis, properties, and purity of the tested nanoparticles was presented in our previous article.^28^ Additionally, the zeta potential (ζ) (mV) of the nanoparticles samples was measured using a laser Doppler electrophoresis (LDE) analyser (Zetasizer Nano ZS ZEN3600, red laser (λ=633 nm), Malvern Instruments Ltd, Malvern, UK). Measurements were performed according to ISO 13099–2:2012 with the following parameters: temperature 22°C, 6 measurements, temperature stabilisation time 120 s, monomodal analysis model, Smoluchowski calculation model (F(ka) value: 1.50). Tests were performed in DTS1070 cuvettes using Zetasizer 7.12 software (Malvern Instruments Ltd., Malvern, UK). For the LDE analysis the ZnONPs suspension sample (100 mL, 15 ppm) was obtained by adding dry ZnONPs powder to deionised water (specific conductance below 0.1 μS/cm, HLP 20UV, Hydrolab, Straszyn, Poland) and then subjected to ultrasonic homogenisation (120 seconds, Elmasonic S 10, 30 W, Germany). The AuNPs (100 ppm) and AgNPs (100 ppm) suspension samples were diluted to a concentration of 15 ppm using deionised water. The zeta potential for ZnONPs was 20.0±0.1 mV, for AgNPs −18.7±2.9 mV, and for AuNPs −23.3±3.1 mV.

The cultures were maintained in a growth room as also described by Kulus et al.^28^

After one week, shoot tips were excised and encapsulated in a 3% sodium alginate solution based on MS medium salts without CaCl_2_, supplemented with 9% sucrose. Beads (3–4 mm in diameter) with a single explant were hardened in 0.1 M CaCl_2_ for 30 min, rinsed, osmoprotected with a loading solution (2.0 M glycerol and 0.4 M sucrose), and dehydrated with Plant Vitrification Solution 3 (PVS3) for 150 min. Ten beads were placed in a 2.0 mL sterile cryovial and immersed in LN.

After a day in LN storage, cryovials were rapidly rewarmed in a water bath (39°C), and explants were rinsed in liquid MS medium with 1.2 M sucrose and inoculated on the MS recovery medium with 3% sucrose and 2.22 μM 6-benzyladenine. A flowchart of key cryopreservation steps is given in Supplementary Figure 1. The cultures were grown in vitro for 60 days as described elsewhere.^28^ Next, the microshoots were rooted on an MS medium with 11.42 μM indole-3-acetic acid for three weeks and acclimatized to ex vitro conditions in a glasshouse following Kulus et al.^30^

### Experiment II – NPs in the Alginate Beads

This experiment included the same cryopreservation steps and parameters as the first one, except for the absence of NPs in the preculture medium. Instead, silver (AgNPs), gold (AuNPs), and zinc oxide (ZnONPs) nanoparticles at concentrations of 5 ppm and 15 ppm were introduced into the sodium alginate solution during encapsulation.

### Histological Analysis, Visualisation of NPs Penetration Into Plant Cells, and Ultrastructure Observation With Transmission Electron Microscopy (TEM)

In vitro-derived shoot tips, 7-day-old, were harvested and fixed in 2.5% glutaraldehyde in 0.1M cacodylate buffer pH=7.2 (all provided by SERVA, Heidelberg, Germany) overnight at room temperature. Samples were washed in cacodylate buffer three times and stained with 1% osmium tetroxide (Sigma-Aldrich) in ddH_2_O overnight at room temperature. Samples were washed in ddH_2_O and dehydrated through a graded series of EtOH (30%, 50%, 70%, 80%, 96%, absolute ethanol and acetone) (Chempur, Piekary Śląskie, Poland). Samples were embedded in epon resin (SERVA) and polymerized 24h at 60°C in incubator (Agar Scientific, Stansted, UK). Next, 400 nm and 70 nm sections were cut with a diamond knife on RMC MTXL ultramicrotome (RMC Boeckeler Instruments, Tucson, AZ, USA) to light and transmission electron microscopy. The sections on a copper grids were analysed in a LIBRA 120 transmission electron microscope (Carl Zeiss, Oberkochen, Germany), at 120 keV. Photographs were made with a Slow-Scan CCD camera (ProScan, Oberkochen, Germany), using the EsiVision Pro 3.2 software. Measurements were performed using the analySIS^®^ 3.0 image-analytical software (Soft Imaging Systems, Muenster, GmbH). Transverse sections to be investigated under light microscope were made from the same blocks and colored by methylene blue (Sigma-Aldrich) in 60°C. Prepared slides were studied and photographed under Evolution 300 light microscope, equipped with camera DLT-Cam PRO (Delta Optical, Warsaw, Poland).

### Genetic Stability Evaluation

The genetic fidelity of 208 in vitro-grown shoots (8 plants from each of the 26 experimental treatments) was assessed using randomly amplified polymorphic DNA (RAPD) and start codon target polymorphism (SCoT) markers.

Total genomic DNA was isolated from fresh leaf tissues using a Genomic Mini AX Plant Spin kit (A&A Biotechnology, Gadńsk Poland), according to the manufacturer’s instruction. The isolated DNA was stored at −80°C in a laboratory deep-freezer. DNA concentration and purity was monitored with the NanoPhotometer NP80 (Implen, München, Germany).

A total of 16 primers (8 RAPD and 8 SCoT) were used for the PCR reaction. Each 25 µL reaction volume contained 2 mm MgCl_2_ in reaction buffer; 1 mm dNTP solution mix; 1 µM single primer; 0.05 U·µL^−1^ Taq DNA polymerase, 0.8 ng·µL^−1^ template DNA (20 ng), and molecular water to volume. RAPD amplification was performed in a C1000 Touch thermal cycler (Bio-Rad, Hercules, CA, USA) programmed as follows: one cycle of 4 min at 94°C for initial DNA denaturation; 40 cycles of 1 min at 94°C for denaturation, 40 sec at 42°C for annealing, and 2 min at 72°C for DNA extension. The last cycle was followed by a final extension step of 4 min at 72°C. As for the SCoT analysis, the following profile was applied: one cycle of 4 min at 94°C for initial DNA denaturation; 35 cycles of 1 min at 94°C for denaturation, 50 sec at 44°C for annealing, and 2 min at 72°C for DNA extension. The last cycle was followed by a final extension step of 8 min at 72°C.

The amplified DNA fragments were separated on 1.5% (*w/v*) agarose gel, DN- and RNase-free, in a TBE buffer at 110 V for 90 min (Biometra P25, Jena, Germany), and detected by staining with ethidium bromide (BrEt). Gel images were recorded using a GelDoc XR+ Gel Photodocumentation System (Bio-Rad) UV transilluminator with Image Lab 4.1 software. Molecular weights of the fragments were estimated using a 100–5,000 bp DNA molecular marker.

The banding patterns were recorded as 0–1 binary matrix, where “1” indicates the presence and “0” the absence of a given fragment. For every primer tested the total number of bands, monomorphic, polymorphic (present in the electrophoretic profile of more than one individual) and specific/unique *loci* (present in the electrophoretic profile of a single individual) were counted.

### Determination of Total Protein Content in Microshoots

In vitro-derived shoot samples (100 mg of fresh weight) were homogenized in phosphate buffer (pH 7.4) containing 1 mm dithiothreitol (DTT), 1 mm EDTA, and 2% polyvinylpyrrolidone (PVP) according to Homaee and Ehsanpour.^31^ The homogenates were centrifuged and supernatants were used to determine the total protein content based on the Bradford^32^ method with bovine serum albumin as the standard (all provided by Sigma-Aldrich). The spectrophotometric analysis was performed at 595 nm in the NanoPhotometer NP80. Three replications were included in each experimental treatment.

### Determination of Antioxidant Capacity of Microshoots

A total of 1 g of air-dried microshoots was used in the antioxidant capacity arrays. Six plants from each experimental treatment were used.

Determination of the antioxidant capacity of in vitro-derived shoots by the FRAP (Ferric Reducing Antioxidant Power) method was conducted following Benzie and Strain.^33^ Immediately before the assay, a FRAP working solution was prepared, ie 250 mL of acetate buffer with a pH of 3.6, 25 mL of the 2,4,6-Tri(2-pyridyl)-s-triazine solution (TPTZ, 10 millimoles in 40 mmol HCl) and 25 mL of iron (III) chloride hexahydrate solution (20 mmol) were mixed (all provided by Sigma-Aldrich). The solution was incubated at 37 °C and assays were then performed. For this purpose, 6 mL of the FRAP solution was taken and 200 μL of the sample and 600 μL of H_2_O were added. After 4 min, absorbance was measured at a wavelength of 593 nm. Based on the conducted measurements, a curve of dependence of the absorbance value on the sample concentration was plotted. Based on the curve, the absorbance value was determined at a concentration equal to the mean of the dilutions used and the antioxidant capacity was calculated at the same absorbance value based on the standard curve determined for Fe^2+^ ions. To remove solid parts, the samples were centrifuged for 5 min on a Rotina 420R centrifuge (Hettich, Vlotho, Germany) at 2250× *g* RCF before the assay.

The antiradical properties of the tested microshoot samples were determined using the DPPH (2,2-diphenyl-1-picrylhydrazyl) radical test at a wavelength of λ = 515 nm according to the methodology developed by Sánchez-Moreno^34^ with the modification consisting in determining the frequency of measurements and selecting the optimal concentration of the ethanolic DPPH solution. The antioxidant activity of the samples was expressed as the percentage of the quenched DPPH radical after incubation with the tested sample for a specific time (t = 30 min) against the control sample.

The percentage of reduction DPPH was calculated using the formula:

$$DPPH{\mathrm{ }} = {\mathrm{ }}{A_{DPPH}}-{\mathrm{ }}{A_t}/{\mathrm{ }}{A_{DPPH}} \times {\mathrm{ }}100{\mathrm{ }}\left[\rm % \right]$$

Where:

A_DPPH_ – absorbance of the control sample,

A_t_ – absorbance of the tested sample after a specified time (t = 30 min).

The measurements were performed twice.

### Determination of Total Phenolic Compounds in Plants

A total of 1 g of dried plant material (obtained from six plants) was weighed and the samples were soaked in 2M NaOH (1:20) and left for 24 hours at room temperature. The extract was centrifuged for 15 minutes at 3000 rpm. Subsequently, the supernatant was decanted and 6M HCl was added dropwise to achieve a pH of 2.5. The mixture was centrifuged again and the supernatant was pipetted off. The solution was further filtered through a 0.45 µm PVDF syringe filter. The extract was diluted at a ratio of 1:2 and analyzed to determine the total content of phenolic compounds. The chromatographic analysis was performed using an HPLC Series 200 liquid chromatograph (PerkinElmer, Waltham, MA, USA) equipped with a DAD detector, with detection at λ_ex_/λ_em_ = 270/330 nm. A Velocity STR analytical column (Bionacom, Coventry, UK) with a particle size of 5 µm and dimensions of 250×4.6 mm I.D. was used. The mobile phase consisted of:

eluent A: H_2_O:CH_3_CN:CH_3_COOH in the ratio of 88.5:10:1.5 (% v/v)

eluent B: CH₃CN

The injection volume was 10 µL, and the analysis duration was 44 minutes. A gradient separation program was applied, with a flow rate of 1.3 mL·min^−1^. The identification of phenolic compounds was based on the chromatogram profile of a standard phenolic compound solution. Quantitative analysis of the identified phenolic compounds was carried out using standard curves of peak area versus phenolic compound concentration (µg·mL^−1^). The measurements were performed in three replications.

### Statistical Analysis

The experiments were set up in a completely randomized design for two cultivars independently. Each experiment, focusing on nanoparticle application either during preculture (prec) or encapsulation (enc) step, included seven treatments: control, 5 ppm AgNPs, 15 ppm AgNPs, 5 ppm AuNPs, 15 ppm AuNPs, 5 ppm ZnONPs, and 15 ppm ZnONPs. Statistical analysis was carried out using one-way ANOVA, followed by mean comparisons using Duncan’s Test (P ≤ 0.05). The linear correlation between the concentration of various phenolic compounds was measured using the Pearson correlation coefficient with Statistica 12.0 (StatSoft, Caracow, Poland).

Agglomerative Hierarchical Clustering with Unweighted Pair-Group Average Method (AHC UPGMA) was used to draw the dendrograms. GenAlex 6.5 software^35^ was used to perform the principal component analysis (PCoA). Population groups were distinguished based on the analysis of molecular variance (AMOVA) estimates with the assumption that NPs-treated and control plants are two separate groups. Values of Heterozygosity index (H), Polymorphic Information Content (PIC), Effective multiplex ratio (E), Marker Index (MI), Discriminating power (D), and Resolving power (R) were also investigated for every primer and marker system used.^36^

## Results

The study on the effects of gold, silver, and zinc oxide nanoparticles on cryopreserved plant material showed that these NPs can accumulate within various compartments of plant cells. While minor genetic changes were observed, the nanoparticles significantly influenced the metabolic profile, particularly affecting the synthesis of phenolic acids and aldehydes, as well as antioxidant mechanisms.

### Histological and Micro-Structural Observations of LN-Treated Explants

**Figure 1:**
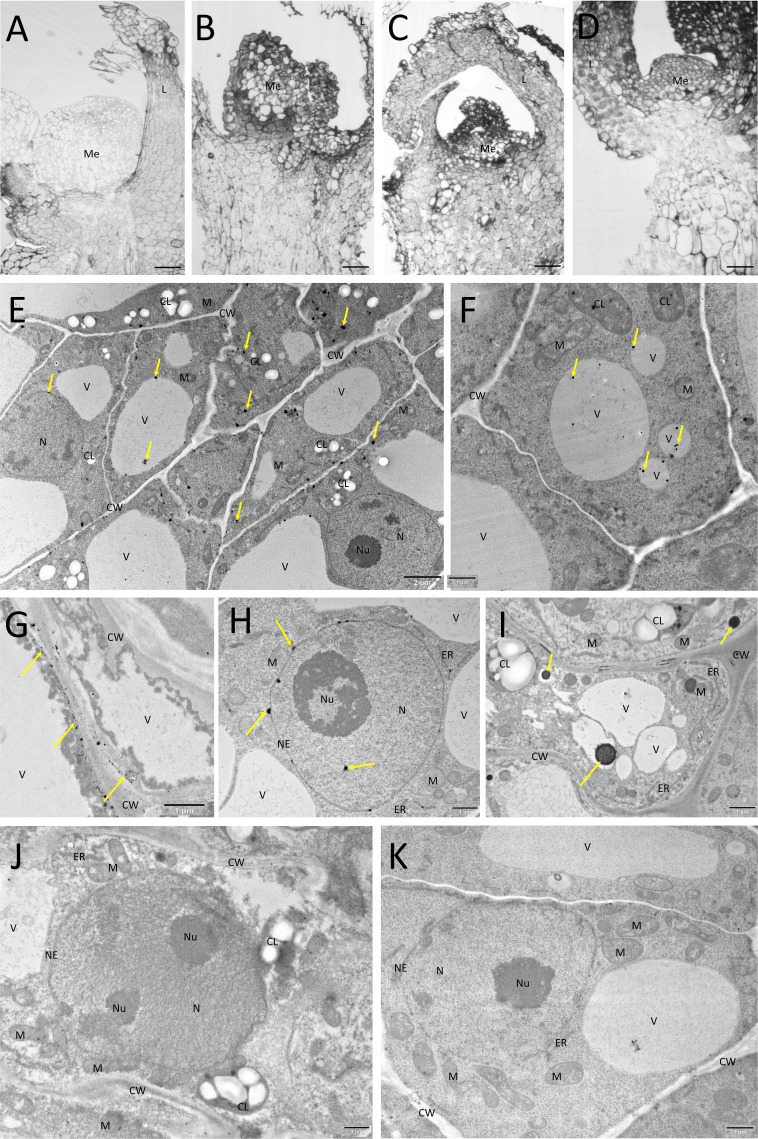
Semi-thin sections and TEM images of LN-derived explants of *L.* *spectabilis* ‘Gold Heart’ after 7 days of recovery culture. (**A**) semi-thin section of a control shoot tip, (**B**–**D**) semi-thin sections of shoot tips treated with AuNPs, AgNPs and ZnONPs, respectively (Me – meristem, L – leaf primordium), Bar = 50µm; The ultrastructure of meristematic cells after incubation with nanoparticles, (**E**) AuNPs visible in various compartments of the plant cell, (**F**) AgNPs in the vacuoles, (**G** and **H**) AgNPs in the cytoplasm just below the cell wall and nucleus, (**I**) ZnONPs forming large aggregates in the cell, (**J** and **K**) control cells without NPs. Nanoparticles are indicated with arrows.

**Abbreviations**: CL, chloroplast; CW, cell wall; ER, endoplasmic reticulum; M, mitochondrion; N, nucleus; NE, nuclear envelope; Nu, nucleolus.

Explants that survived cryopreservation were found to have generally intact meristems (tunica and several layers of corpus cells). Viable cells were also found in leaf primordia (Figure 1A). A slight deformation of the meristem was observed when Au and Ag nanoparticles were applied (Figure 1B and C). The living cells had a typical structure with signs of further development (Figure 1D). TEM analysis confirmed that all types of nanoparticles used in the study were able to penetrate into the cells. Gold nanoparticles were found mainly in the cytoplasm and vacuoles (Figure 1E) Silver nanoparticles accumulated in the cytoplasm near the cell wall, nuclear envelope and inside the cell nucleus (Figure 1F–H). Zinc oxide nanoparticles were localized in the cytoplasm and vacuoles (Figure 1I). No structure changes were found in the cells of the control shoot tips (Figure 1J and K).

### Effect of Nanoparticles on the Genetic Stability of LN-Derived Plants

A total of 15,934 and 15,810 scorable bands were detected by RAPD and SCoT primers in 104 ‘Gold Heart’ and 104 ‘Valentine’ plants, respectively (Table 1). SCoTs generated more products and polymorphisms: two *loci* were polymorphic in a total of 12 ‘Gold Heart’ specimens, detected in equal numbers by primers S-4 and S-6. No polymorphisms were detected by this marker system in bleeding heart ‘Valentine’. As for this cultivar, only one RAPD primer (R-6) detected one polymorphic band in two samples. No polymorphism were detected by RAPDs in the ‘Gold Heart’ cultivar (Table 1).

**Table 1:** Comparative Analysis of Molecular Products Obtained From *L.* *spectabilis* ‘Gold Heart’ (GH) and ‘Valentine’ (V) Plants Analyzed With RAPD and SCoT Markers

Primer Code	Primer Sequence 5′ ➔ 3′	Total no. of Bands		No. of *loci*								No. and (%) of Plants with Polymorphism		No. of Genotypes	
				Total		Mono.		Poly.		Spec.					
		GH	V	GH	V	GH	V	GH	V	GH	V	GH	V	GH	V
**RAPD**															
R-1	GGG AAT TCG G	624	520	6	5	6	5	0	0	0	0	0	0	1	1
R-2	GAC CGC TTG T	936	936	9	9	9	9	0	0	0	0	0	0	1	1
R-3	GGA CTG GAG T	728	728	7	7	7	7	0	0	0	0	0	0	1	1
R-4	GCT GCC TCA GC	936	936	9	9	9	9	0	0	0	0	0	0	1	1
R-5	TAC CCA GGA GCG	1040	1040	9	10	9	10	0	0	0	0	0	0	1	1
R-6	CAA TCG CCG T	1144	1146	11	12	11	11	0	1	0	0	0	2(1.9)	1	2
R-7	GGT GAC GCA G	1040	1040	10	10	10	10	0	0	0	0	0	0	1	1
R-8	CCC AGT CAC T	416	416	4	4	4	4	0	0	0	0	0	0	1	1
**∑**		6864	6762	65	66	65	65	0	1	0	0	0	2(1.9)	1	2
**(mean)**		(858)	(845.2)	(8.1)	(8.2)	(8.1)	(8.1)	(0)	(0.1)	0	(0)	-	-	-	-
**SCoT**															
S-1	CAA CAA TGG CTA CCA CCG	1248	1248	12	12	12	12	0	0	0	0	0	0	1	1
S-2	CAA CAA TGG CTA CCA CCT	1352	1352	13	13	13	13	0	0	0	0	0	0	1	1
S-3	CAA CAA TGG CTA CCA CGT	1352	1352	13	13	13	13	0	0	0	0	0	0	1	1
S-4	ACG ACA TGG CGA CCA ACG	1155	1144	12	11	11	11	1	0	0	0	11(10.6)	0	2	1
S-5	ACC ATG GCT ACC ACC GTC	1144	1144	11	11	11	11	0	0	0	0	0	0	1	1
S-6	ACC ATG GCT ACC ACC GTG	1155	1144	12	11	11	11	1	0	0	0	11(10.6)	0	2	1
S-7	CCA TGG CTA CCA CCG CCA	1040	1040	10	10	10	10	0	0	0	0	0	0	1	1
S-8	CCA TGG CTA CCA CCG CAG	624	624	6	6	6	6	0	0	0	0	0	0	1	1
**∑**		9070	9048	89	87	87	87	2	0	0	0	12(11.5)	0	4	1
**(mean)**		(1133.8)	(1131)	(11.1)	(10.9)	(10.9)	(10.9)	(0.2)	(0)	(0)	(0)	-	-	-	-

**Abbreviations**: RAPD, Randomly Amplified Polymorphic DNA; SCoT, Start Codon Target Polymorphism; Mono, monomorphic; Poly, polymorphic, Spec, specific (unique; present in a single band profile).

Among the two marker systems that detected polymorphic bands, the highest mean H, PIC, E, Mi and R values were reported with SCoTs (Table 2). However, when comparing individual primers, the highest values of H, PIC and D indices were found in R-6 primer (Table 2).

**Table 2:** Values of Heterozygosity Index (H), Polymorphic Information Content (PIC), Effective Multiplex Ratio (E), Marker Index (MI), Discriminating Power (D), and Resolving Power (R) of the Marker Systems Used in the Study

Primer	H		PIC		E		MI		D		R	
Cultivar	GH	V	GH	V	GH	V	GH	V	GH	V	GH	V
	**RAPD**											
R-1	0	0	0	0	0	0	0	0	0	0	0	0
R-2	0	0	0	0	0	0	0	0	0	0	0	0
R-3	0	0	0	0	0	0	0	0	0	0	0	0
R-4	0	0	0	0	0	0	0	0	0	0	0	0
R-5	0	0	0	0	0	0	0	0	0	0	0	0
R-6	0	0.150	0	0.139	0	11.019	0	0.001	0	0.157	0	0.038
R-7	0	0	0	0	0	0	0	0	0	0	0	0
R-8	0	0	0	0	0	0	0	0	0	0	0	0
**Mean**	0	0.019	0	0.017	0	1.377	0	0.000	0	0.020	0	0.005
	**SCoT**											
S-1	0	0	0	0	0	0	0	0	0	0	0	0
S-2	0	0	0	0	0	0	0	0	0	0	0	0
S-3	0	0	0	0	0	0	0	0	0	0	0	0
S-4	0.138	0	0.128	0	11.106	0	0.001	0	0.144	0	0.212	0
S-5	0	0	0	0	0	0	0	0	0	0	0	0
S-6	0.138	0	0.128	0	11.106	0	0.001	0	0.144	0	0.212	0
S-7	0	0	0	0	0	0	0	0	0	0	0	0
S-8	0	0	0	0	0	0	0	0	0	0	0	0
**Mean**	0.035	0	0.032	0	2.777	0	0.000	0	0.036	0	0.053	0

**Abbreviations**: RAPD, Randomly Amplified Polymorphic DNA; SCoT, Start Codon Target Polymorphism.

**Figure 2:**
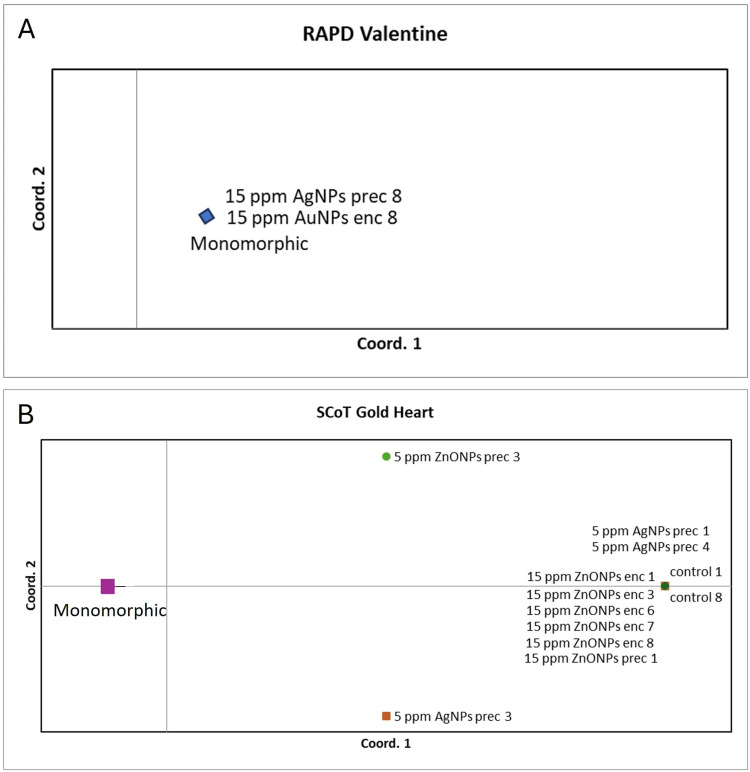
Graphs of principal coordinates analysis (PCoA) of *L. spectabilis* ‘Valentine’ (**A**) and ‘Gold Heart’ (**B**) individuals after various treatments; based on Randomly Amplified Polymorphic DNA (RAPD) and Start Codon Target Polymorphism (Scot) markers.

**Figure 3:**
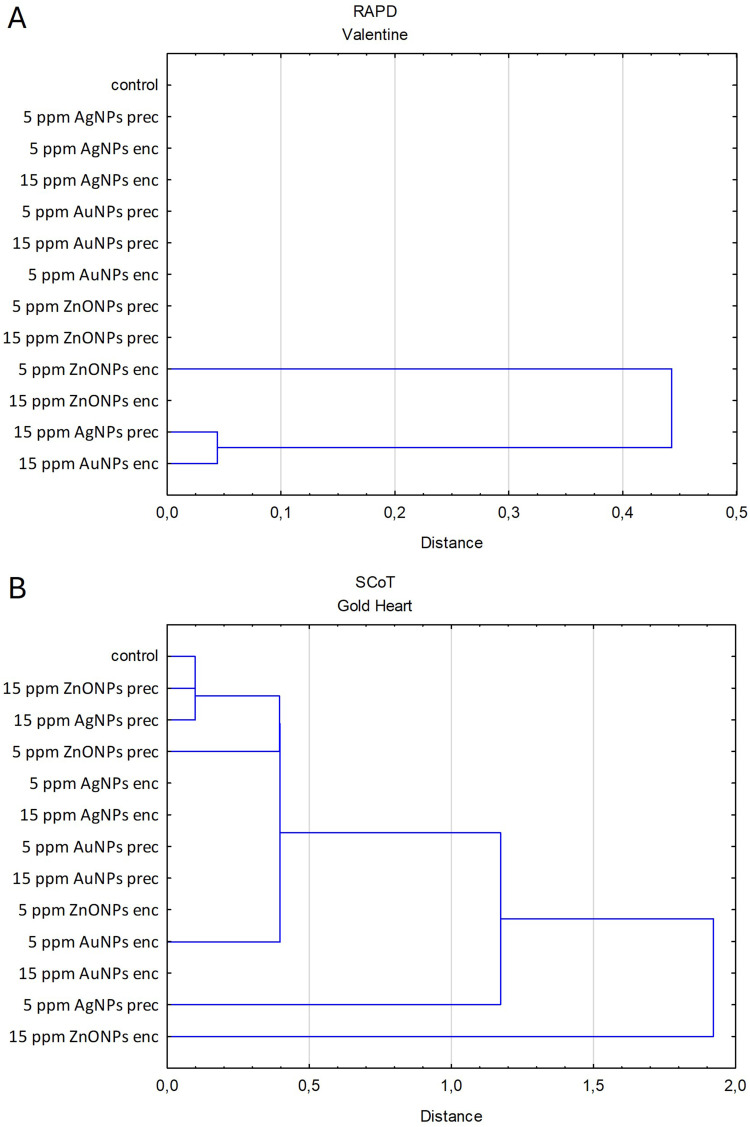
Dendrograms based on the Euclid genetic distance matrix and UPGMA clustering presenting the relationships between the entire plant populations of *L. spectabilis* ‘Valentine’ (**A**) and ‘Gold Heart’ (**B**) after various treatments.

The PCoA analysis based on RAPD genotyping showed that all ‘Valentine’ plants were grouped into a single agglomeration due to minor genetic variation detected in one specimen treated with 15 ppm AgNPs in the preculture medium and one with 15 ppm AuNPs in the alginate bead (Figure 2A). These populations were clustered together in the UPGMA analysis with a small distance from other groups (Figure 3A). For ‘Gold Heart’, four genotypes were identified, leading to four agglomerations in the PCoA array (Figure 2B). Polymorphic specimens were found in both control and nanoparticle treatments. The most distant population from the control consisted of plants treated with 15 ppm ZnONPs. Two other populations included single plants treated with 5 ppm AgNPs or ZnONPs during preculture. No polymorphisms were found in bleeding heart ‘Gold Heart’ after using AuNPs (either in the preculture or encapsulation steps) or AgNPs (in the encapsulation step). UPGMA analysis revealed two major clusters for ‘Gold Heart’, with one including most experimental groups and the other consisting of plants treated with 15 ppm ZnONPs (Figure 3B).

**Figure 4:**
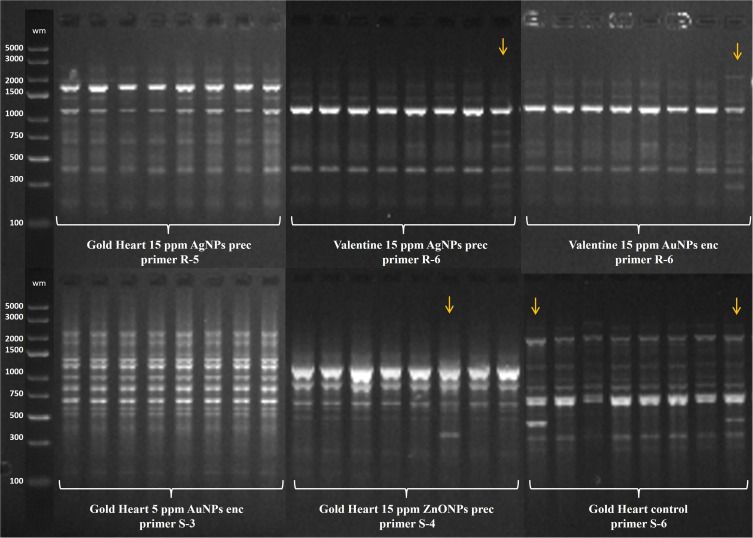
Example RAPD (R) and Scot (S) band profiles of *L. spectabilis* ‘Gold Heart’ and ‘Valentine’ received as a result of nanoparticles treatment. Outermost lanes (wm) are DNA bp weight markers, while inner lines represent plants treated with gold (AuNPs), silver (AgNPs) or zinc oxide (ZnONPs) nanoparticles added either into the preculture medium (prec) or alginate bead (enc). Arrows indicate polymorphic genotypes.

Following the AMOVA analysis, the entire genetic variation detected by the RAPD markers in bleeding heart ‘Valentine’ was of intra-population origin, ie there was no significant effect of nanoparticle treatments. As for ‘Gold Heart’ cultivar, it was found that 94% of the variation detected by SCoTs was of within-population origin, while 6% was caused by nanoparticles (Table 3). Example band profiles generated by RAPD and SCoT markers are shown in Figure 4.

**Table 3:** Analysis of Molecular Variance (AMOVA)

Source of Variation	df	SS	MS	Est. Var.	%
	**RAPD Valentine**				
**Among populations**	12	0.212	0.018	0.000	0%
**Within populations**	91	1.750	0.019	0.019	100%
**Total**	103	1.962		0.019	100%
	**SCoT Gold Heart**				
**Among populations**	1	0.361	0.361	0.012	6%
**Within populations**	102	19.313	0.189	0.189	94%
**Total**	103	19.673		0.201	100%

**Notes**: AMOVA results for 208 plants, based on standard permutation across the full data set.

**Abbreviations**: df, degrees of freedom; SS, sum of squares; MS, mean square; Est. Var, estimated variance; %, proportion of genetic variability.

### Effect of Nanoparticles on the Protein Content in the LN-Derived Plants

**Figure 5:**
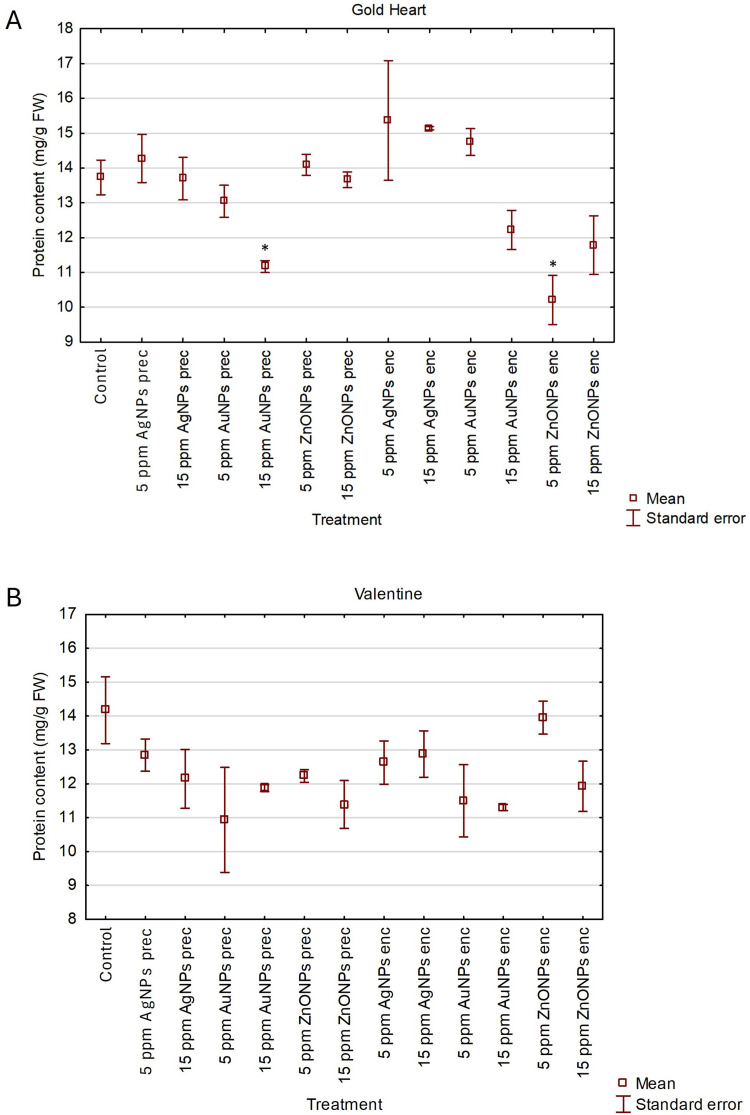
Effect of silver (AgNPs), gold (AuNPs), and zinc oxide (ZnONPs) nanoparticles applied during the preculture (prec) or encapsulation (enc) step of the encapsulation-vitrification cryopreservation protocol on the total protein content in shoots after 60 days of recovery culture in *L. spectabilis* ‘Gold Heart’ (**A**) and ‘Valentine’ (**B**). Mean values (± standard errors) marked with an asterisk (*) differ significantly from the control according to Duncan’s post hoc test (P<0.05).

None of the NPs-treated plants of bleeding heart ‘Gold Heart’ synthesized more proteins than the untreated control (13.73 mg·g^−1^ FW). However, supplementation of preculture medium with 15 ppm AuNPs and alginate matrix with 5 ppm ZnONPs resulted in a significantly lower protein content (10.21–11.17 mg·g^−1^ FW). As for the ‘Valentine’ cultivar, there was no effect on nanoparticles on the protein synthesis (10.93–13.95 mg·g^−1^ FW) compared with the control (14.17 mg·g^−1^ FW) (Figure 5A and B).

### Effect of Nanoparticles on the Antioxidant Capacity of LN-Derived Plants

In both cultivars studied, it was found that most of the NPs-treated plants had a higher antioxidant capacity (according to both FRAP and DPPH arrays) than the control (Table 4). Lower FRAP and DPPH values were found only in the treatments with 5 and 15 ppm ZnONPs in the preculture medium (except for the DPPH test in bleeding heart ‘Gold Heart’, in which the lowest value was reported in the control). On the other hand, the highest FRAP value was found when 15 ppm gold or zinc oxide nanoparticles were added into the alginate matrix (for ‘Gold Heart’ and ‘Valentine’, respectively). As for the DPPH array, the highest values were found in the treatments: 15 ppm AgNPs in the alginate matrix or 15 ppm AuNPs in the preculture medium for ‘Gold Heart’ and ‘Valentine’ cultivars, respectively.

**Table 4:** Effect of Silver (AgNPs), Gold (AuNPs), and Zinc Oxide (ZnONPs) Nanoparticles Applied During the Preculture (Prec) or Encapsulation (Enc) Step of the Encapsulation-Vitrification Cryopreservation Protocol on the Antioxidant Capacity of Microshoots (FRAP and DPPH) After 60 days of Recovery Culture in *L. spectabilis* ‘Gold Heart’ and ‘Valentine’

	FRAP (µmol Fe^2+^·100mL^−1^)				DPPH (%)							
**Treatment**	**Gold Heart**											
Control	3716.6	±	0.41	k		54.85	±		0.05		k	
5 ppm AgNPs prec	5127.2	±	0.21	j		80.10	±		0.40		i	
15 ppm AgNPs prec	7622.4	±	8.30	e		89.10	±		0.01		d	
5 ppm AuNPs prec	7378.4	±	1.26	g		86.05	±		0.05		f	
15 ppm AuNPs prec	8073.5	±	0.85	b		93.60	±		0.01		c	
5 ppm ZnONPs prec	2315.2	±	1.04	l		82.90	±		0.01		h	
15 ppm ZnONPs prec	2641.9	±	0.81	m		87.25	±		0.05		e	
5 ppm AgNPs enc	7467.4	±	0.60	f		84.70	±		0.10		g	
15 ppm AgNPs enc	7845.3	±	0.85	c		98.30	±		0.01		a	
5 ppm AuNPs enc	7266.6	±	0.61	h		76.30	±		0.10		j	
15 ppm AuNPs enc	8480.5	±	0.41	a		83.25	±		0.05		h	
5 ppm ZnONPs enc	7112.6	±	0.23	i		93.35	±		0.05		c	
15 ppm ZnONPs enc	7686.1	±	0.61	d		94.50	±		0.10		b	
	**Valentine**											
Control	1063.5	±	0.82	l		44.90		±		0.10		h
5 ppm AgNPs prec	1441.9	±	0.81	j		88.05		±		0.05		b
15 ppm AgNPs prec	2715.6	±	1.44	h		80.25		±		0.05		e
5 ppm AuNPs prec	5102.9	±	0.81	d		48.80		±		0.10		g
15 ppm AuNPs prec	6301.3	±	1.25	b		88.35		±		0.05		a
5 ppm ZnONPs prec	844.2	±	1.89	m		30.25		±		0.15		j
15 ppm ZnONPs prec	1208.9	±	0.60	k		41.90		±		0.10		i
5 ppm AgNPs enc	2682.4	±	1.46	i		77.55		±		0.05		f
15 ppm AgNPs enc	3607.1	±	0.84	g		80.10		±		0.10		e
5 ppm AuNPs enc	4700.0	±	0.40	f		85.25		±		0.05		d
15 ppm AuNPs enc	4949.2	±	0.63	e		87.85		±		0.05		b
5 ppm ZnONPs enc	5200.6	±	1.05	c		77.55		±		0.05		f
15 ppm ZnONPs enc	9174.7	±	0.82	a		87.20		±		0.10		c

**Notes**: Each number represents the mean value ± standard error. Significant differences in values are determined by Duncan’s post hoc test (*P*<0.05). Values with at least one same letter are not statistically different.

### Effect of Nanoparticles on the Polyphenols Content in the LN-Derived Plants

**Figure 6:**
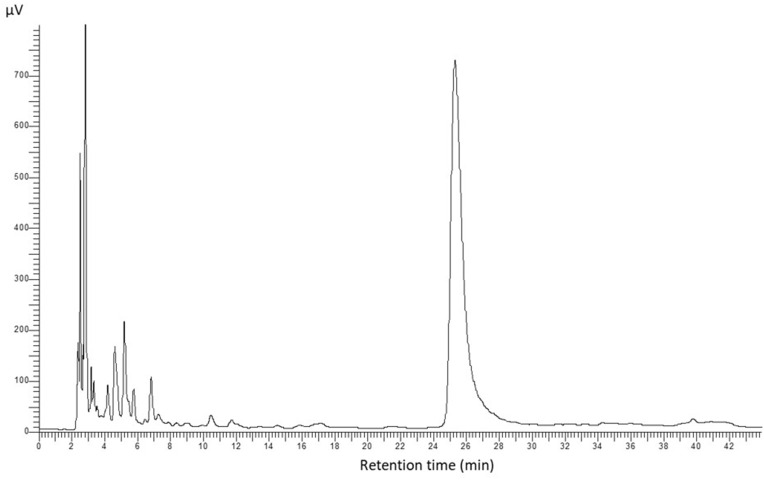
An example chromatogram of *L. spectabilis* ‘Valentine’ sample obtained from the treatment 15 ppm AgNPs in the encapsulation bead matrix: protocatechuic acid (5 min), vanillic acid (11 min), syringic acid (12 min), vanillin aldehyde (15 min), syringaldehyde (18 min), p-coumaric acid (24 min).

The application of nanoparticles had a significant impact on the metabolic profile of bleeding heart plants (Table 5, Figure 6). The addition of AuNPs either into the preculture medium or encapsulation bead or ZnONPs into the alginate bead increased the synthesis of protocatechuic acid in ‘Gold Heart’ plants. In turn, the presence of AgNPs in the alginate had a deleterious effect compared with the control. On the other hand, the addition of AgNPs into the preculture medium, ZnONPs into the alginate (both regardless of concentration) and 15 ppm AuNPs into the alginate increased the concentration of vanillic acid, whereas AuNPs, ZnONPs in the preculture medium and AgNPs in the alginate matrix had a negative effect. The content of syringic acid in ‘Gold Heart’ cultivar was increased by most treatments, except for ZnONPs in the preculture medium. In contrast, most of the NP-treatments, except AgNPs, inhibited the production of vanillic aldehyde. None of the treatments suppressed the production of syringaldehyde, some NPs even had a positive effect on this parameter. In contrast, the production of p-coumaric acid was inhibited by the presence of 5 ppm AuNPs or AgNPs in the preculture medium or alginate beads, respectively (Table 5). Consequently, ‘Gold Heart’ plants from the experimental objects: 5 ppm AuNPs/ZnONPs in the preculture medium and 5 ppm AgNPs in the alginate matrix had a reduced total phenolics content (7866.6–8778.9 µg·g^−1^ DW) compared with the untreated control (11577.1 µg·g^−1^ DW).

**Table 5:** Effect of Silver (AgNPs), Gold (AuNPs), and Zinc Oxide (ZnONPs) Nanoparticles Applied During the Preculture (Prec) or Encapsulation (Enc) Step of the Encapsulation-Vitrification Cryopreservation Protocol on the Concentration of Various Phenolic Compounds After 90 days of Ex Vitro Growth in *L. spectabilis* ‘Gold Heart’ and ‘Valentine’

	Protocatechuic acid (µg·g^−1^ DW)							Vanillic acid (µg·g^−1^ DW)							Syringic acid (µg·g^−1^ DW)							Vanillic aldehyde (µg·g^−1^ DW)						
Treatment	Gold Heart																											
Control	1997.5	±		19.6		f		345.3		±	2.6		c		281.6		±	4.8		e	154.1				±	4.8		ab
5 ppm AgNPs prec	2085.6	±		40.5		d-f		452.0		±	7.4		a		341.9		±	5.8		c	146.9				±	3.3		bc
15 ppm AgNPs prec	2050.9	±		51.1		ef		438.5		±	9.8		a		334.3		±	5.4		c	140.9				±	3.5		c
5 ppm AuNPs prec	2213.3	±		109.3		de		186.6		±	13.3		e		386.3		±	9.5		b	156.4				±	0.6		a
15 ppm AuNPs prec	2843.6	±		8.6		b		188.0		±	1.6		e		483.9		±	3.2		a	126.9				±	0.8		d
5 ppm ZnONPs prec	1286.3	±		6.2		i		119.7		±	2.4		f		202.6		±	3.0		gh	109.3				±	0.5		e
15 ppm ZnONPs prec	2036.9	±		41.5		ef		158.6		±	5.5		e		225.4		±	5.8		f	121.7				±	1.8		d
5 ppm AgNPs enc	1552.1	±		1.8		h		99.7		±	1.9		f		178.7		±	4.2		h	153.5				±	1.1		ab
15 ppm AgNPs enc	1777.1	±		66.3		g		273.7		±	2.6		d		309.1		±	4.1		d	147.6				±	0.8		bc
5 ppm AuNPs enc	2481.9	±		10.6		c		323.6		±	8.2		c		410.8		±	12.8		b	122.5				±	1.0		d
15 ppm AuNPs enc	3070.8	±		69.5		a		463.8		±	6.0		a		342.6		±	9.1		c	114.5				±	1.2		e
5 ppm ZnONPs enc	2555.7	±		5.1		c		406.9		±	6.7		b		388.9		±	16.7		b	145.2				±	2.4		c
15 ppm ZnONPs enc	2249.2	±		129.9		d		399.5		±	30.6		b		400.3		±	12.1		b	143.8				±	3.4		c
	**Valentine**																											
Control	1259.9	±		19.1		i		177.8		±	8.0		de		189.5	±		5.2		ef	115.7				±	1.3		ef
5 ppm AgNPs prec	2008.8	±		56.4		cd		139.2		±	8.8		fg		195.6	±		3.6		ef	137.0				±	0.4		b
15 ppm AgNPs prec	1842.8	±		3.0		ef		156.6		±	2.9		ef		223.3	±		3.1		bc	116.7				±	0.3		e
5 ppm AuNPs prec	2208.0	±		39.9		b		188.6		±	3.4		d		282.8	±		16.2		a	128.0				±	0.8		cd
15 ppm AuNPs prec	1775.5	±		2.9		g		122.5		±	6.2		g		231.7	±		3.9		b	140.1				±	0.7		b
5 ppm ZnONPs prec	2067.0	±		49.4		c		178.9		±	4.3		de		185.7	±		1.7		ef	128.7				±	0.6		cd
15 ppm ZnONPs prec	1911.3	±		4.2		de		164.9		±	4.2		e		178.6	±		8.4		f	124.9				±	1.4		d
5 ppm AgNPs enc	1466.1	±		18.2		h		309.4		±	7.9		b		217.2	±		2.4		bc	116.3				±	2.1		ef
15 ppm AgNPs enc	1452.9	±		6.5		h		291.8		±	3.9		b		140.6	±		2.4		g	111.5				±	1.6		fg
5 ppm AuNPs enc	1389.6	±		71.7		h		339.2		±	4.4		a		180.1	±		9.5		f	131.4				±	2.0		c
15 ppm AuNPs enc	962.4	±		6.7		j		174.4		±	2.1		de		137.0	±		5.9		g	107.4				±	0.7		g
5 ppm ZnONPs enc	2303.4	±		21.6		b		175.1		±	16.7		de		202.8	±		8.7		cd	130.4				±	3.7		c
15 ppm ZnONPs enc	3657.1	±		66.7		a		231.9		±	2.0		c		286.4	±		5.6		a	145.7				±	1.0		a
	**Syringaldehyde (µg·g^−1^ DW)**							**p-Coumaric acid (µg·g^−1^ DW)**								**Total phenolics (µg·g^−1^ DW)**												
**Treatment**	**Gold Heart**																											
Control	124.7		±		1.2		d		8673.9			±		79.6		a-c			11577.1				±	100.1			ab	
5 ppm AgNPs prec	241.5		±		23.2		a		8479.7			±		22.9		a-c			11747.6				±	86.4			ab	
15 ppm AgNPs prec	140.3		±		6.6		cd		7255.4			±		201.9		b-d			10360.2				±	257.7			bc	
5 ppm AuNPs prec	181.9		±		10.6		b		5654.3			±		2330.9		d			8778.9				±	2356.4			cd	
15 ppm AuNPs prec	255.8		±		7.4		a		8411.5			±		293.8		a-c			12309.7				±	277.1			ab	
5 ppm ZnONPs prec	156.2		±		3.3		b-d		6734.1			±		85.9		cd			8608.2				±	97.0			cd	
15 ppm ZnONPs prec	141.2		±		10.4		cd		10083.7			±		246.7		a			12767.5				±	258.7			a	
5 ppm AgNPs enc	160.4		±		6.4		b-d		5722.3			±		19.1		d			7866.6				±	11.3			d	
15 ppm AgNPs enc	271.5		±		15.6		a		9162.8			±		4.6		ab			11941.8				±	60.7			ab	
5 ppm AuNPs enc	266.3		±		5.9		a		7611.9			±		61.0		b-d			11216.9				±	56.8			ab	
15 ppm AuNPs enc	148.3		±		7.3		b-d		8336.0			±		48.4		a-c			12476.0				±	63.9			ab	
5 ppm ZnONPs enc	137.6		±		5.4		cd		7323.7			±		125.1		b-d			10958.1				±	140.5			ab	
15 ppm ZnONPs enc	168.0		±		19.2		bc		8267.1			±		26.8		a-c			11627.8				±	198.9			ab	
	**Valentine**																											
Control	236.1		±		16.7		b		8440.4			±		71.0		c			10419.3				±	37.5			e	
5 ppm AgNPs prec	169.2		±		10.4		de		6655.8			±		247.4		g			9305.7				±	298.8			g	
15 ppm AgNPs prec	134.2		±		11.2		f		8766.4			±		48.0		c			11240.0				±	48.0			d	
5 ppm AuNPs prec	158.1		±		5.1		d-f		7086.5			±		156.9		f			10052.1				±	185.9			ef	
15 ppm AuNPs prec	153.4		±		0.8		ef		7671.1			±		172.2		d			10094.2				±	176.0			ef	
5 ppm ZnONPs prec	151.6		±		3.6		ef		7576.1			±		108.7		de			10288.0				±	156.1			e	
15 ppm ZnONPs prec	148.2		±		5.4		ef		7249.8			±		106.9		ef			9777.7				±	93.6			f	
5 ppm AgNPs enc	204.1		±		7.1		c		11898.6			±		103.4		a			14211.7				±	127.4			a	
15 ppm AgNPs enc	156.6		±		7.0		d-f		10733.3			±		69.6		b			12886.7				±	67.2			b	
5 ppm AuNPs enc	186.3		±		19.7		cd		10612.8			±		111.2		b			12839.5				±	195.3			b	
15 ppm AuNPs enc	128.3		±		1.2		f		7369.5			±		57.9		d-f			8878.9				±	59.8			g	
5 ppm ZnONPs enc	175.1		±		8.0		c-e		7294.6			±		96.7		d-f			10281.4				±	79.0			e	
15 ppm ZnONPs enc	538.3		±		8.4		a		7455.0			±		77.7		d-f			12314.4				±	146.1			c	

**Notes**: Each number represents the mean value ± standard error. Significant differences in values are determined by Duncan’s post hoc test (*P*<0.05). Values with at least one same letter are not statistically different.

In the case of *L. spectabilis* ‘Valentine’, most of the experimental treatments stimulated the biosynthesis of protocatechuic acid and vanillic aldehyde, except for 15 ppm AuNPs in the alginate bead, which had a deleterious effect (Table 5). The addition of 5/15 ppm AgNPs, 5 ppm AuNPs and 15 ppm ZnONPs into the alginate matrix enhanced the production of vanillic acid, p-coumaric acid and total phenolics (also 15 ppm AgNPs in the preculture medium in the case of total phenolics). Supplementation of alginate with a higher (15 ppm) concentration of Ag or AuNPs suppressed the production of syringic acid, although it was elevated by several other treatments. Conversely, nearly all ‘Valentine’ plants had a lowered syringaldehyde content compared with the control, except for those from the experimental object 15 ppm ZnONPs in the alginate matrix, which contained a higher concentration of this compound (Table 5).

A positive correlation (0.44–0.76) between the content of protocatechuic acid and vanillic or syringic acids, as well as vanillic acid and syringic acid or syringic acid and syringaldehyde was found in bleeding heart ‘Gold Heart’ (Table 6). No significant negative correlations between the concentrations of phenolic compounds were reported in this cultivar. On the other hand, there was a strong positive correlation (0.72–0.84) between the content of protocatechuic acid and syringic acid, vanillin aldehyde, syringaldehyde, and between vanillic acid and p-coumaric acid in the ‘Valentine’ cultivar. A moderate positive correlation (0.48–0.59) was reported between syringic acid and vanillin aldehyde/syringaldehyde or syringaldehyde and vanillin aldehyde. Contrarily, there was a negative correlation between the concentration of protocatechuic acid and p-coumaric acid (−0.41) in the ‘Valentine’ plants.

**Table 6:** Correlation Between the Concentration of Various Phenolic Compounds After 90 days of Ex Vitro Growth in *L. spectabilis* ‘Gold Heart’ and ‘Valentine’

Compound	Protocatechuic Acid	Vanillic Acid	Syringic Acid	Vanillin Aldehyde	Syringaldehyde	p-Coumaric Acid
	**Gold Heart/Valentine**					
Protocatechuic acid	1.00/1.00	0.51*/-0.13	0.76*/0.72*	−0.20/0.73*	0.15/0.73*	0.20/-0.41*
Vanillic acid	0.51*/-013	1.00/1.00	0.44*/-0.14	0.10/-0.18	−0.04/0.21	0.23/0.84*
Syringic acid	0.76*/0.72*	0.44*/-0.14	1.00/1.00	0.04/0.59*	0.45*/0.53*	0.07/-0.24
Vanillin aldehyde	−0.20/0.73*	0.10/-0.18	0.04/0.59*	1.00/1.00	−0.02/0.48*	−0.20/-0.41*
Syringaldehyde	0.15/0.73*	−0.04/0.21	0.45*/0.53*	−0.02/0.48*	1.00/1.00	0.15/-0.06
p-Coumaric acid	0.20/-0.41*	0.23/0.84*	0.07/0.24	−0.20/-0.41*	0.15/-0.06	1.00/1.00

**Notes**: Correlation coefficients marked with an asterisk (*) are significant at p<0.05.

## Discussion

### Genetic Stability of LN-Recovered Plant Material

The number of scorable bands detected by both RAPD and SCoT markers indicates the genomic coverage and efficiency of each marker system.^37^ In this study, SCoT markers produced more bands compared to RAPDs, in both cultivars studied, proving a higher resolution and discriminatory power. Moreover, the higher frequency of polymorphisms detected by SCoT markers (and higher values of diversity indices: H, PIC, E, Mi, and R), especially in ‘Gold Heart’ plants, indicated a greater ability to capture genetic variation within the studied species. This was due to the targeted nature of SCoT markers, which amplify specific regions of the genome, compared to the random nature of RAPD markers.^38^

According to the AMOVA analysis, a non-significant variation was found within two ‘Valentine’ plants in the present study. As for the ‘Gold Heart’ cultivar, it was estimated that 6% of the total variation detected (in 10.5% of all plants analyzed) was related to NPs treatment, especially the presence of 15 ppm ZnONPs in the alginate beads. The TEM-detected presence of nanoparticles in the nucleus justified the observed genetic variation. Due to their lower solubility and a general tendency to sediment, ZnONPs formed larger aggregates in the cells compared to AgNPs or AuNPs. In a study on purslane (*Portulaca oleracea* L)., ZnONPs were found to affect cell ultrastructure, causing changes in intracellular organelles such as chloroplasts, which became less consistent and dilated.^39^ Additionally, ZnO NPs formed aggregates of around 120 nm in root cells, confirming their tendency to aggregate within plant tissues.^40^ This explains why most of the somaclones in the present study were detected after the application of this particular nanoparticle type. The genotoxic effects of NPs have been described previously.^41^ Nanoparticles can induce mutations directly or indirectly. Direct DNA damage can occur through physical interactions between NPs and DNA molecules, leading to strand breaks, cross-linking, or base modifications. Indirect DNA damage may result from the generation of reactive oxygen species (ROS) by NPs, affecting both DNA bases and the 2-deoxyribose component of the DNA backbone.^42^ Murali et al^43^ mentioned the genotoxic and cytotoxic properties of ZnONPs on onion (*Allium cepa* L.) root tips. Likewise, random amplified polymorphic DNA assays confirmed ZnONPs-induced changes in the DNA structure of buckwheat (*Fagopyrum esculentum* Moench).^44^ These reports explained the obtained-here variation. Despite cryopreservation is consider a nearly ideal strategy for the long-term preservation of plant genetic resources, ROS-induced oxidative stresses were documented to induce (epi)genetic and somatic variations in LN-derived plants, even without the use of nanoparticles.^27^ Also in the present study, polymorphic plants were detected within the non-NP-treated ‘Gold Heart’ control. This led us to the conclusion that this cultivar is particularly susceptible to somaclonal variation occurrence due to the presence of some hotspot regions in its genome.^45^ This hypothesis is supported by the fact that most of the polymorphic plants had the same band profile (ie mutations occurred in the same places). In future studies, it would be interesting to identify these hotspots for breeding purposes. On the other hand, the ‘Valentine’ cultivar seems more genetically stable.

### Effect of Nanoparticles on the Metabolic Activity of LN-Derived Bleeding Heart

The results suggest that nanoparticles have varying effects on metabolic activity in different cultivars of bleeding heart. In ‘Gold Heart’, the supplementation of preculture medium with 15 ppm AuNPs and alginate matrix with 5 ppm ZnONPs resulted in significantly lower protein content, suggesting a potential inhibitory effect of these NPs on protein synthesis. Conversely, Mehmood and Murtaza^21^ and Pandey et al^46^ reported that in seeds of *Pisum sativum* L. and *Brassica juncea* L., AgNPs stimulated the synthesis of proteins and carbohydrates. As for the ‘Valentine’ cultivar, there was no significant effect of NPs on protein synthesis compared to the control, confirming the cultivar-specific action of nanoparticles.^47^

Nanoparticles might directly interact with genes, enzymes and intermediates involved in the biosynthesis of phenolic compounds, leading to their activation or stabilization. It can be suggested that NPs induce changes in the expression levels of genes associated with the phenylpropanoid pathway, which is the primary metabolic route for the synthesis of phenolic acids and aldehydes.^48^,^49^ Upregulation of PAL (phenylalanine ammonia lyase), C4H (cinnamate-4-hydroxylase), and 4CL (4-coumarate-CoA ligase) genes justify the spectacular increase of protocatechuic and syringic acid concentrations in most NPs treatments, in both cultivars studied. Likewise, pharmaceutically important phenolic compounds (xanthomicrol and cirsimaritin) overproduction and enhanced expression of *pal* and *ras* genes were reported in *Dracocephalum kotschyi* Boiss. hairy roots elicited by SiO_2_ nanoparticles.^50^ On the other hand, NPs might bind to and inhibit key enzymes in these pathways, leading to a decrease in phenolic compound synthesis, depending on the specific circumstances and characteristics of the nanoparticles involved,^51^ for example in the case of vanillic and syringaldehyde aldehydes in ‘Valentine’ cultivar. Nonetheless, the elevated content of some phenolics in favorable conditions, particularly evident in the ‘Valentine’ cultivar treated with NPs during the encapsulation step, can be very beneficial for the pharmacological industry, since compounds such as protocatechuic acid are prized for their anti-inflammatory, antihyperglycemic and antiapoptotic activities,^52^ opening new research prospects in plant biotechnology.

The final effect of NPs depends on various factors, including their shape, size, concentration, coatings, self-aggregate tendency, and stability. While some nanoparticles have been shown to exhibit antioxidant properties, others may induce the production of ROS, which can lead to oxidative stress.^53^ Increased values of FRAP and DPPH in most NPs-treated combinations indicate stronger activity of redox-active antioxidants, meaning it is more effective at scavenging free radicals and protecting against oxidative stress-related damage compared with the non-treated control.^54^ Nanoparticles can penetrate plant cells effectively, increasing the availability of antioxidants and facilitating their interaction with free radicals, hence increasing the FRAP and DPPH values.^55^ Thus, it can be concluded that the NPs-treated plants are adapting to stress by boosting their defenses against oxidative damage. For example, AuNPs synthesized through biological methods have been shown to exhibit good antioxidant activity. They can scavenge various types of radicals, including hydroxyl radicals and nitric oxide radicals, which are harmful to biological systems.^56^ In contrast, the low values of FRAP and DPPH indices after adding ZnONPs into the preculture medium or alginate bead suggest a lower level of oxidative stress and dormancy of antioxidant mechanisms.^57^ These results correspond with the lowered content of anthocyanins and flavonoids in the leaves of ex-vitro-grown ‘Gold Heart’ plants, reported previously. On the other hand, ZnONPs-supplementation of preculture medium stimulated the biosynthesis of chlorophyll and enhanced photosynthesis in this cultivar further corresponding with the overall better quality of the plants. These findings are in agreement with the study of Geremew^58^ on mustard (*Brassica juncea* (L). Czern). Zinc is an essential micronutrient for plants, regulating the function of many enzymes and hormones, as well as macromolecule metabolism, stabilizing protein structures and affecting gene expression.^59^

This study explored the effects of gold, silver, and zinc oxide nanoparticles on cryopreserved bleeding heart plants, finding that nanoparticles can accumulate within cells, induce minor genetic changes, and significantly influence metabolic profiles, particularly affecting phenolic acids and antioxidant mechanisms.

## Conclusion

This study highlights the potential of nanoparticles in enhancing plant cryopreservation techniques, offering innovative solutions for preserving plant genetic resources on the brink of environmental challenges. Nanoparticles can penetrate the meristematic cell of bleeding heart and accumulate in its organelles, including the cell wall, cytoplasm, vacuoles and nucleus. Zinc oxide NPs tend to form particularly big aggregates. Thus, NP treatments may cause minor genetic variation in LN-derived plants. This effect is more evident in ‘Gold Heart’ plants than in ‘Valentine’, highlighting the potential for NPs to induce genetic changes in a cultivar-specific manner. The synthesis of proteins is less dependent on NPs treatment, but the application of nanoparticles during cryopreservation has a significant impact on the production of phenolic acids and aldehydes in both ‘Gold Heart’ and ‘Valentine’ cultivars, as well as their antioxidant defense mechanism. These findings can be utilized to modulate plant secondary metabolism and growth for desired pharmacological outcomes. They can also be implemented in the long-term storage of genetic resources valuable in breeding. Overall, our findings underline the cultivar-specific and NP-dependent effects on the metabolic, structural, and genetic stability in bleeding heart plants. Further research is needed to understand the underlying mechanisms and potential long-term impacts on plant physiology and development, such as changes in the levels of pro-healthy alkaloids characteristic of the Papaveraceae family. Additionally, the effect of other types of NPs should be considered.

## Data Availability

All files are available from the RepOD database (accession number https://doi.org/10.18150/NB0TVW).
